# SARS-CoV2 Infection and the Importance of Potassium Balance

**DOI:** 10.3389/fmed.2021.744697

**Published:** 2021-10-27

**Authors:** Helen C. Causton

**Affiliations:** Department of Pathology and Cell Biology, Columbia University Irving Medical Center, New York, NY, United States

**Keywords:** electrolyte, renin-angiotensin system, drug repurposing, SARS-CoV-2 infection, potassium

## Abstract

SARS-CoV2 infection results in a range of symptoms from mild pneumonia to cardiac arrhythmias, hyperactivation of the immune response, systemic organ failure and death. However, the mechanism of action has been hard to establish. Analysis of symptoms associated with COVID-19, the activity of repurposed drugs associated with lower death rates or antiviral activity *in vitro* and a small number of studies describing interventions, point to the importance of electrolyte, and particularly potassium, homeostasis at both the cellular, and systemic level. Elevated urinary loss of potassium is associated with disease severity, and the response to electrolyte replenishment correlates with progression toward recovery. These findings suggest possible diagnostic opportunities and therapeutic interventions. They provide insights into comorbidities and mechanisms associated with infection by SARS-CoV2 and other RNA viruses that target the ACE2 receptor, and/or activate cytokine-mediated immune responses in a potassium-dependent manner.

## Introduction

SARS-CoV2 infects cells *via* interaction with the ACE2 receptor which is found primarily on the surface of the heart, liver, kidney, and lungs (1). ACE2 is a negative regulator of the renin-angiotensin system (RAS) that acts in conjunction with ion transporters and the insulin receptor to protect against hypertension, diabetes, cardiovascular disease, and organ damage (2). It does this by regulating electrolyte balance and blood pressure, cell volume, intercellular signaling, filtering of urine in the kidney, membrane potential, and the firing rate of electrically active cells (3, 4). Binding of ACE2 by the SARS-CoV2 virus and the processes of viral entry and replication, enhance degradation of the receptor, which decreases inhibition of the classical RAS system. The net result is increased reabsorption of sodium and water, and raised blood pressure (5). Hypokalemia/low intracellular potassium can also lead to cellular hyperpolarity, increased resting potential, and depolarization in cardiac and lung cells that can trigger ventricular arrhythmia and respiratory dysfunction (6). In parallel, expression of the viral viroporin, Orf3a protein actively promotes potassium efflux, and stimulates activation of the innate immune response. It does so by triggering the cell-intrinsic Nod-like receptor family, pyrin domain-containing 3 (NLRP3) inflammasome (7–9), which promotes cytokine release. Inflammasome responses play fundamental roles in clearing viruses and promoting tissue repair (10), however, hyperactivation of this immune response, gives rise to the devastating “cytokine storm” that is associated with severe infection, and a major cause of death (11).

This mini-perspective discusses the effects of electrolyte and potassium imbalance in SARS-CoV2 infection, describes how a number of comorbidities of COVID-19 affect ion homeostasis and, identifies some drugs effective against SARS-CoV2 *in vivo* that have also been shown to affect pH or K+ balance. Collectively, these findings highlight the importance of maintaining, and promoting electrolyte homeostasis. They also provide a framework for beginning to understand the broad, and seemingly unrelated, range of symptoms associated with COVID-19 and possibly other RNA viruses, that target the ACE2 receptor and/or those that activate the NRPL3 inflammasome in a potassium-dependent manner.

## Potassium Imbalance is Common Among Patients With Severe SARS-CoV2 Infection

Potassium homeostasis is maintained at a systemic level, in the balance between dietary intake (~100 mmol/day) and excretion (95% *via* the kidney; 5% *via* the colon) and *via* internal balance of K^+^ between intracellular and extracellular fluid compartments (4). Hypokalemia, typically defined as <3.5 mmol/L in plasma, shares many of the features of SARS-CoV2 infection, including muscle weakness, palpitations, cardiac dysrhythmias, and poor diabetic control (4, 12).

In the course of SARS-CoV2 infection, hypokalemia is primarily caused by elevated aldosterone, which promotes excretion of potassium in urine (13). One study involving 1,415 patients, found electrolyte imbalance and hypokalemia were associated with disease severity (Weighted Mean Difference:0.12 mmol/L [95% CI: −0.18 to −0.07 mmol/ L], I21/433%) (14). Another found that hypokalemia around the time of admission was associated with a requirement for invasive mechanical ventilation (15), while a smaller study observed that although only 54% of the patients (*n* = 175) had low potassium levels, of the severely ill patients 85% had hypokalemia (13). A case-controlled study of three emergency rooms in France found that hypokalemia and hyponatremia (sodium <135 mmol/L) were independently associated with COVID-19 infection, but that low sodium, and not potassium levels were associated with ICU admission (16). Disease severity is also related to the degree of response to potassium replacement as mildly ill COVID-19 patients with hypokalemia in the Chen study achieved normokalemia within 5–8 days of potassium replacement (3 g potassium chloride or 40 mEq/day), whereas, it took 10–14 days to achieve homeostasis potassium in severely ill patients (13). Severe hypokalemia may be harder to correct as it is associated with alkalosis (29% had a ≥ pH 7.45) (13). This is due to hydrogen-potassium exchange between the intra and extracellular fluid (4). Patients with COVID-19 are also susceptible to pro-arrhythmic effects (17).

## A Number of Comorbidities for COVID-19 Affect Ion Homeostasis

Patients with severe symptoms of COVID-19 are more likely to have kidney or cardiovascular disease, hypertension, diabetes mellitus (DM) or other comorbidities than those with milder symptoms (18–22). The association between COVID-19 and a number of these comorbidities is bidirectional (23, 24): patients with diabetes are more likely to develop severe symptoms or die of COVID-19 (12, 22) and acute diabetes or acid-ketosis can develop as a result of SARS-CoV2 infection (25–28). High levels of insulin are found in the olfactory bulb in the brain. Insulin modulates the voltage-dependent potassium channel, Kv1.3, and suppresses the Kv1.3-contributed current in cultured olfactory bulb neurons (OBNs) of rodents (29, 30),while deletion of the Kv1.3 channel results in “super smeller” mice (31). There is little data on the effect of decreased insulin production on the Kv1.3 channel, however it may contribute to the anosmia experienced by some COVID-19 patients (32).

## A Number of Repurposed Drugs Effective Against SARS-CoV2 Affect Potassium Balance

It has been hard to obtain insights into the mechanism by which SARS-CoV2 acts, based on the diversity of symptoms identified in infected individuals. Likewise, FDA approved drugs that act *in vitro* to reduce viral replication and plaque formation, increase cell viability, or are associated with lower death rates in patients target a range of host factors. These drugs are used for a wide range of purposes from treatment of malaria to pancreatitis and diabetes (33–36) (Table 1). However, some patterns are emerging: 17 of 66 FDA approved drugs with anti-viral activity were found to target the Sigma-1 receptor (σ1-R) and sigma-2 receptor (σ2-R) (SIGMAR1/SIGMAR2) (34). Sigma receptors are ubiquitously expressed in mammalian tissues and are involved in cellular signaling in a number of conditions including retinal and neurodegenerative disorders (37, 38). A number of σ1-R and σ2-R receptor agonists have been found to inhibit Kv2.1 potassium channel activity in a receptor-independent manner (39), suggesting that they act to modulate potassium currents directly. Another 7 of the 69 drugs inhibit protein synthesis (34). Although the mechanism is not known, protein synthesis, and potassium abundance are inversely correlated in systems as diverse as yeast, algae, and mouse fibroblasts (40–43), such that inhibition of protein synthesis would be expected to result in greater intracellular potassium abundance. A further 17 drugs have been shown to affect osmotic or ion homeostasis. Agonists of potassium channels, angiotensin II, and protein synthesis were also found to be enriched among drugs with anti-SARS-CoV2 activity in an independent study (35).

**Table 1:** Repurposed drugs with anti-viral activity that also affect potassium balance.

**Drug**	**Human target**	**Anti-viral activity**	**Indication**	**Affects**	**Reference**
Camostat	Cell Entry	(44, 45)	Pancreatitis	Elevates Na+:K+ ratio	(46)
Chloroquine	Cell Entry	(47)	Malaria, immune modulation	Blocks hERG K+ channels	(48)
Hydroxy chloroquine	Cell Entry	(34, 47)	Malaria, immune modulation	Blocks hERG K+ channels	(48)
Loratadine	SLC6A15	(49)	Antihistamine	Kv1.5, outward current	(50, 51)
Nafamostat	Cell Entry	(52)	Pancreatitis	Can induce hyperkalemia, by suppressing the Na-K ATPase dependent pathway	(53)
Pioglitazone	CISD1	(54)	Diabetes	Remodeling of Kv1.5 & Kv4.2	(55)
YH-1238	H+, K+ ATPase Proton Pump	(35)	Phase I	H^+^,K^+^-ATPase (ATP4A, ATP4B)	(35)

Some of these repurposed drugs many act to reduce disease severity *via* their effects on the immune system. Sex hormones, such as progesterone, promote immune tolerance, and anti-inflammatory responses and that may account for lower COVID-related disease severity and mortality in women and during pregnancy (56, 57). Clinical studies of drug efficacy also point to the key role of the renin-angiotensin system and electrolyte balance in influencing patient outcomes. A retrospective study of COVID-19 patients taking famotidine, an antiacid, found that hospitalized patients taking the drug were more than twice as likely to survive (33). Famotidine was also identified in a computational screen of drugs likely to have anti-SARS-CoV2 activity (36). Another drug, Nafamostat, acts on potassium balance by reducing urinary excretion of potassium *via* the Na+/K+ ATPase-dependent pathway (58, 59). These data support the idea that restoring potassium balance promotes a better host response against viral infection. Conversely some of these drugs pose a risk as they promote hyperkalemia (48, 60). This is a complication found in a number of patients who die of COVID-19 (37% of those who died (n = 113) compared with 14% (*n* = 161) of those who recovered (61).

Potassium dysregulation is also likely to form part of the mechanism that promotes viral pathogenicity. A study that ectopically expressed the SARS-CoV2 envelope (E) protein in HEK 293 and NIH3T3 cells found that it formed a pH-dependent ion channel permeable to potassium and sodium ions (62). Only a small proportion of the E protein ends up in the viral envelope and most is localized to the endoplasmic reticulum-Golgi complex where it multimerizes to form a virioporin, that promotes an increase in intra-golgi pH (62, 63). The E protein channel is critical for infectivity and for the pathogenicity of SARS-CoV2, as it is for other coronaviruses, and thus presents a good target for therapeutic intervention (63, 64).

## Discussion

Taken together, these observations drawn from comorbidities, clinical features of disease and the possible targets of drugs that are effective against viral infection show that symptoms associated with low intracellular potassium are similar to those that result from SARS-CoV2 infection, and that potassium efflux can promote hyperactivation of the innate immune response. Although we do not yet understand how SARS-CoV2 acts in detail, potassium balance is likely to be important for both the propagation and pathogenicity of the virus, *via* effects on both the virus, and on homeostatic mechanisms in the host.

It is likely that this line of enquiry will have relevance for understanding the consequences of viral infection more broadly. Ion disturbance, mediated by virioporins, is central to the mechanism of action of a range of viruses from influenza, and rhinovirus to COVID-19 and HIV (8), and a number of RNA viruses modulate activity of the NLRP3 inflammasome in a potassium-dependent manner (65, 66). In bats, dampening of the inflammasome and proinflammatory responses confers tolerance to a range of RNA viruses, suggesting that modulating the inflammasome may prove a useful therapeutic target for reducing disease severity in humans too (10).

Similarities between SARS-CoV2 and other coronaviruses offer further mechanistic insight and opportunities for drug repurposing. SARS-CoV1 also enters the cell *via* the ACE2 receptor and can cause acute lung failure, cardiac arrhythmia, gastrointestinal disorders, hyperkalemia and diabetes (4, 5, 67, 68). Nafamostat, which induces hyperkalemia, inhibits the activity of SARS-CoV1, 2 and MERS-CoV (52, 53, 60, 69). Approximately 50 FDA-approved drugs are known to have activity against all 3 viruses (70). These results present a strong argument for gaining a fundamental understanding of how electrolyte balance functions in both the healthy host and in response to viral infection. This knowledge is expected to identify strategies for diagnosis and therapeutic intervention in patients suffering from a number of virally induced diseases.

## Data Availability Statement

The original contributions presented in the study are included in the article/supplementary material, further inquiries can be directed to the corresponding author/s.

## Author Contributions

The author confirms being the sole contributor of this work and has approved it for publication.

## Conflict of Interest

The author declares that the research was conducted in the absence of any commercial or financial relationships that could be construed as a potential conflict of interest.

## Publisher's Note

All claims expressed in this article are solely those of the authors and do not necessarily represent those of their affiliated organizations, or those of the publisher, the editors and the reviewers. Any product that may be evaluated in this article, or claim that may be made by its manufacturer, is not guaranteed or endorsed by the publisher.
